# Sleep-related changes in astrocytic biomarkers are modulated by *APOE* ε4 genotype in cognitively unimpaired adults

**DOI:** 10.1093/braincomms/fcaf437

**Published:** 2025-11-07

**Authors:** Nuole Zhu, Miren Altuna, Javier Arranz, Íñigo Rodriguez-Baz, Maria Belén Sanchez-Saudinós, Laura Videla, Sílvia Valldeneu, Mireia Carrera-Vega, Sergio Romero, Juan Fortea, Alberto Lleó, Sandra Giménez, Daniel Alcolea

**Affiliations:** Sant Pau Memory Unit, Department of Neurology, IR SANT PAU, Hospital de la Santa Creu i Sant Pau, Barcelona 08025, Spain; Centro de Investigación Biomédica en Red en Enfermedades Neurodegenerativas (CIBERNED), Madrid 28029, Spain; Faculty of Medicine, Universitat Autònoma de Barcelona, Barcelona 08193, Spain; CITA-Alzheimer Foundation, Donostia-San Sebastián 20009, Spain; Osakidetza, Organización Sanitaria Integrada (OSI), Debabarrena 20850, Spain; Sant Pau Memory Unit, Department of Neurology, IR SANT PAU, Hospital de la Santa Creu i Sant Pau, Barcelona 08025, Spain; Faculty of Medicine, Universitat Autònoma de Barcelona, Barcelona 08193, Spain; Sant Pau Memory Unit, Department of Neurology, IR SANT PAU, Hospital de la Santa Creu i Sant Pau, Barcelona 08025, Spain; Centro de Investigación Biomédica en Red en Enfermedades Neurodegenerativas (CIBERNED), Madrid 28029, Spain; Sant Pau Memory Unit, Department of Neurology, IR SANT PAU, Hospital de la Santa Creu i Sant Pau, Barcelona 08025, Spain; Centro de Investigación Biomédica en Red en Enfermedades Neurodegenerativas (CIBERNED), Madrid 28029, Spain; Sant Pau Memory Unit, Department of Neurology, IR SANT PAU, Hospital de la Santa Creu i Sant Pau, Barcelona 08025, Spain; Centro de Investigación Biomédica en Red en Enfermedades Neurodegenerativas (CIBERNED), Madrid 28029, Spain; Barcelona Down Medical Center, Fundació Catalana Síndrome de Down, Barcelona 08041, Spain; Sant Pau Memory Unit, Department of Neurology, IR SANT PAU, Hospital de la Santa Creu i Sant Pau, Barcelona 08025, Spain; Centro de Investigación Biomédica en Red en Enfermedades Neurodegenerativas (CIBERNED), Madrid 28029, Spain; Sant Pau Memory Unit, Department of Neurology, IR SANT PAU, Hospital de la Santa Creu i Sant Pau, Barcelona 08025, Spain; Automatic Control Department (ESAII) and Institute for Research and Innovation in Health (IRIS), Universitat Politècnica de Catalunya-BarcelonaTech (UPC), BarcelonaTech (UPC), Barcelona 08005, Spain; Sant Pau Memory Unit, Department of Neurology, IR SANT PAU, Hospital de la Santa Creu i Sant Pau, Barcelona 08025, Spain; Centro de Investigación Biomédica en Red en Enfermedades Neurodegenerativas (CIBERNED), Madrid 28029, Spain; Barcelona Down Medical Center, Fundació Catalana Síndrome de Down, Barcelona 08041, Spain; Sant Pau Memory Unit, Department of Neurology, IR SANT PAU, Hospital de la Santa Creu i Sant Pau, Barcelona 08025, Spain; Centro de Investigación Biomédica en Red en Enfermedades Neurodegenerativas (CIBERNED), Madrid 28029, Spain; Faculty of Medicine, Universitat Autònoma de Barcelona, Barcelona 08193, Spain; Sant Pau Memory Unit, Department of Neurology, IR SANT PAU, Hospital de la Santa Creu i Sant Pau, Barcelona 08025, Spain; Centro de Investigación Biomédica en Red en Enfermedades Neurodegenerativas (CIBERNED), Madrid 28029, Spain; Multidisciplinary Sleep Unit, Respiratory Department, IR SANT PAU, Hospital de la Santa Creu i Sant Pau, Barcelona 08025, Spain; Sant Pau Memory Unit, Department of Neurology, IR SANT PAU, Hospital de la Santa Creu i Sant Pau, Barcelona 08025, Spain; Centro de Investigación Biomédica en Red en Enfermedades Neurodegenerativas (CIBERNED), Madrid 28029, Spain

**Keywords:** astrocytes, Alzheimer’s disease-related proteins, polysomnography, apolipoprotein ε4, cognitively unimpaired individual

## Abstract

Astrocytes are key regulators of sleep and neuroinflammatory responses. However, the relationship between objective sleep parameters and astrocytic fluid biomarkers in cognitively unimpaired individuals remains unclear. We examined how sleep architecture relates to astrocytic, neuroaxonal and Alzheimer’s disease-related fluid biomarkers in cognitively unimpaired adults and whether age, sex and *APOE* ε4 moderate these associations. This cross-sectional study included 51 cognitively unimpaired participants from the Sant Pau Initiative on Neurodegeneration cohort. One-night in-lab polysomnography was used to quantify sleep architecture, fragmentation, slow-wave activity and respiratory parameters. CSF biomarkers included glial fibrillary acidic protein (GFAP), chitinase-like-3 protein 1 (YKL-40), Aβ42, Aβ40, pTau181 and tTau; plasma biomarkers included GFAP and neurofilament light chain (NfL). Associations were analysed using Spearman correlations, multiple linear regression, and moderation models, adjusting for age, sex, body mass index, *APOE* ε4 status and sleep apnoea. Lighter and more fragmented sleep, characterized by longer N1 duration, increased wake after sleep onset, frequent stage transitions and elevated cortical arousal, was associated with higher CSF YKL-40, Aβ40, pTau181 and tTau (*ρ* = 0.32–0.62, all *P* < 0.05). In contrast, deeper, more consolidated sleep, indicated by longer total time of sleep, greater N3 duration and higher slow-wave activity, was associated with lower CSF GFAP and YKL-40 (*ρ* = −0.35 to −0.44, all *P* < 0.05). These associations remained significant in adjusted regression models. Plasma GFAP and NfL exhibited an inverse profile, with positive associations with deeper sleep (*β*: 0.16–0.18, *P* < 0.05) and negative associations with lighter sleep stages (*β*: −0.23 to −0.29, *P* < 0.01). Rapid eye movement (REM) sleep was also associated with astrocytic fluid biomarkers, with negative correlations for CSF and plasma GFAP (*ρ* = −0.49 and *ρ* = −0.28, respectively, all *P* < 0.05), while in regression models, REM duration remained a negative predictor of plasma GFAP (*β* = −0.23, *P* = 0.003) and a positive predictor of CSF YKL-40 (*β* = 0.12, *P* = 0.037). Notably, *APOE* ε4 consistently moderated associations between sleep and CSF YKL-40 and GFAP, while age and sex influenced plasma GFAP and CSF YKL-40, respectively (all *P* < 0.05). In cognitively unimpaired adults, sleep architecture is differentially associated with central and peripheral biomarkers of astrocytic activation, neuroaxonal integrity and Alzheimer’s disease-related proteins. These findings support the importance of considering sleep as a key factor in the early pathophysiology of neurodegenerative disease.

## Teaser

Zhu *et al*. report that in cognitively unimpaired adults, sleep architecture is differentially associated with CSF and plasma biomarkers of astrocytic activation, neuroaxonal integrity and Alzheimer’s disease-related proteins. These associations were moderated by *APOE* ε4 status, suggesting a distinct pattern in individuals genetically predisposed to Alzheimer’s disease.

## Graphical Abstract

**Graphical Abstract:**
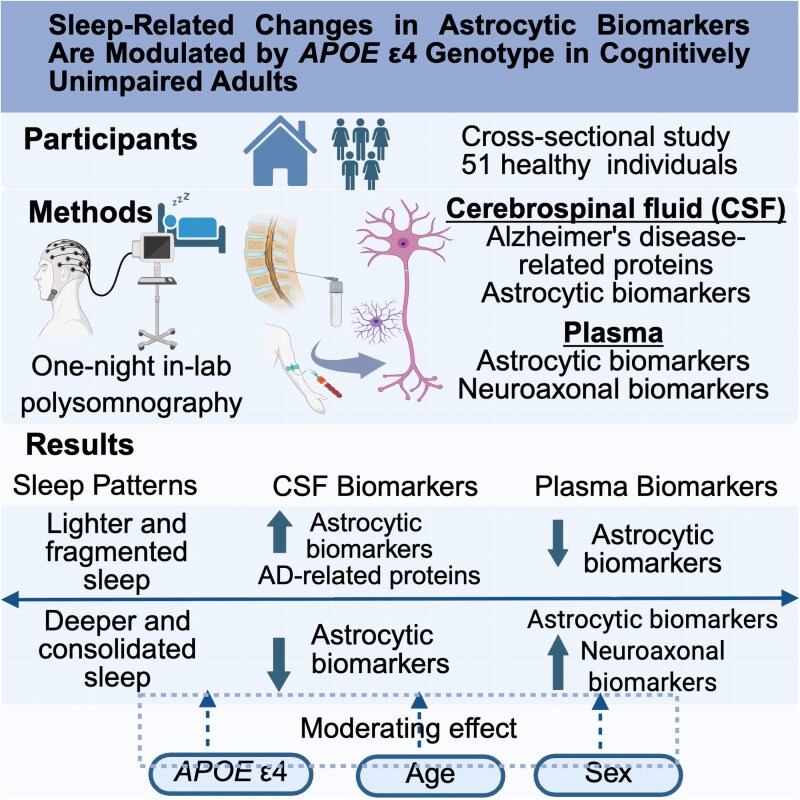

## Introduction

In recent years, increasing efforts have focused on understanding the biological mechanisms that contribute to the pathophysiology and progression of neurodegenerative diseases, with a growing focus on sleep disturbances and neuroinflammation.^1-4^

Sleep disturbances are common in neurodegenerative conditions such as Alzheimer’s disease and are increasingly recognized to interact bidirectionally with disease-related pathology.^1,3,5-14^ Disrupted sleep may exacerbate Alzheimer’s disease pathology by impairing the production and clearance of amyloid and tau proteins, processes that predominantly occur during deep sleep [so-called slow-wave sleep (SWS) or N3 stage of non-rapid eye movement (REM) sleep].^5-7^ Conversely, sleep changes often appear early in the course of the disease, potentially driven by amyloid and tau depositions in brain regions involved in sleep regulation, thereby promoting further neurodegeneration and cognitive decline.^8-12^ Evidence from both subjective and objective sleep assessments has linked greater amyloid burden to shorter sleep duration, reduced sleep efficiency (SE), increased sleep latency (SL) and a higher prevalence of obstructive sleep apnoea (OSA) and daytime napping.^9,13-17^ In addition, lower slow-wave activity (SWA), a specific electrophysiological marker of slow, high-amplitude oscillations during SWS, has also been associated with amyloid and tau pathology in cognitively unimpaired (CU) individuals.^8,10^

Furthermore, neuroinflammation is another key feature of neurodegenerative diseases, including Alzheimer’s disease, and may precede clinical symptoms by years or even decades.^2,4,18,19^ Sustained immune responses in the brain, primarily mediated by activated microglia and astrocytes, are closely linked to neurodegeneration.^2,18,19^ These glial cells have been shown to modulate the clearance and production of Aβ42 and contribute to the development and propagation of tau pathology.^20-22^

Notably, astrocytes participate in sleep regulation by modulating sleep–wake dynamics through intracellular signalling pathways and the release of sleep-inducing molecules.^23,24^ Neuroinflammation has been proposed as a potential link between sleep disturbances and increased risk of neurodegenerative diseases.^25^ Astrocytic reactivity appears to be an early event in Alzheimer’s continuum. Glial fibrillary acidic protein (GFAP) and chitinase-3-like protein (YKL-40) are considered two robust fluid biomarkers of astrocyte activation *in vivo*, and both have been shown to increase in CU, Aβ-positive individuals, prior to overt neurodegeneration or cognitive decline.^26-29^ Elevated serum GFAP concentrations have been reported in individuals with chronic insomnia and to correlate with insomnia severity.^30^ Likewise, increased YKL-40 and neurofilament light chain (NfL), a marker of neuroaxonal injury, in CSF have been associated with poor sleep and worse self-reported sleep quality in both Alzheimer’s disease and non-Alzheimer’s disease individuals.^16,31-33^ Studies in both animals and humans also suggest that sleep disruption may trigger astrocytic activation, contributing to local and systemic inflammatory responses linking poor sleep to Alzheimer’s disease pathology.^25,34,35^

Moreover, sleep disturbances and neuroinflammation also share common risk factors with Alzheimer’s disease, such as apolipoprotein E ε4 (*APOE* ε4) genotype and ageing. The *APOE* ε4 genotype, the strongest genetic risk factor for Alzheimer’s disease, has been associated with reduced sleep quality in CU individuals, based on both subjective reports and objective measures.^36-38^ Experimental studies have shown that *APOE* ε4 exacerbates the effect of sleep disruption on Aβ and tau pathology and alters glial responses.^39^ Although the association between *APOE* ε4 and sleep-disordered breathing remains inconclusive, some studies have reported an increased risk of sleep apnoea in *APOE* ε4 carriers.^40,41^ A large proportion of brain APOE is produced by astrocytes, where it supports lipid metabolism, Aβ clearance and blood–brain barrier (BBB) maintenance. In animal models, expression of the APOE ε4 isoform has been associated with increased astrocytic inflammation and BBB dysfunction.^42-45^

Ageing is associated with chronic low-grade inflammation—often referred to as ‘inflammaging’—which has been linked to poorer sleep quality, including more frequent and prolonged nighttime awakenings.^46-48^ Age-related glial activation has also been associated with sleep microstructure disruption, tau phosphorylation, synaptic loss and memory impairment, even in the absence of β-amyloid positivity.^49^ These converging associations point towards a complex interplay between sleep, neuroinflammation, and Alzheimer’s disease-related processes.

However, our understanding of how sleep interacts with biomarkers relevant to neurodegenerative disease remains limited and is hindered by methodological constraints. Many studies have focused on isolated sleep parameters—ranging from respiratory indices and sleep duration to macro- and microstructural features—each capturing different aspects of sleep physiology.^3,17,50,51^ Others rely on self-reported questionnaires that may not accurately capture the extent of sleep disturbances.^52,53^ In addition, the presence of neurodegenerative disease, which may precede symptom onset by several years, can itself influence sleep–biomarker relationships. Studies that overlook preclinical Alzheimer’s disease risk a misinterpretation, since early pathological processes may already impact sleep patterns and astrocytic activity.^1,3^

Despite increasing interest in sleep–Alzheimer’s disease interactions, few studies examined astrocytic biomarkers in relation to sleep,^18,25,35^ and even fewer have considered how common risk factor such as age and *APOE* ε4 may moderate these associations.

Given the early involvement of astrocytic reactivity and sleep alterations in the Alzheimer’s continuum, and their shared association with common risk factors, it is plausible that sleep–biomarker relationships may already be detectable in CU individuals. Investigating these relationships in a rigorously characterized, biologically defined, disease-free cohort may provide insight into early mechanisms of neurodegenerative vulnerability and how they may be shaped by such factors. This approach offers a physiological reference point against which pathological changes can later be compared.

Therefore, the present study aimed to:

- characterize the associations between objective nocturnal polysomnography (NPSG) macro and microstructure derived sleep parameters and fluid biomarkers of astrocytic activation (CSF GFAP, YKL-40 and plasma GFAP), neuroaxonal injury (plasma NfL) and Alzheimer’s disease-related proteins (CSF Aβ40, Aβ42, pTau181 and tTau) in cognitively unimpaired adults;
- identify independent sleep-related parameters associated with biomarker concentrations, accounting for relevant covariates; and
- explore whether these associations are moderated by age, sex or *APOE* ε4 genotype.

## Materials and methods

### Ethics approval and consent to participate

All participants provided written informed consent. The study was approved by the local ethics committee of Hospital de la Santa Creu I Sant Pau following the principles stated in the Declaration of Helsinki (IIBSP-DOW-2014-30; v5: 11 July 2016, v6: 24 October 2023).

### Participants and study design

Fifty-one CU adult participants from the Sant Pau Initiative on Neurodegeneration (SPIN) cohort who underwent one-night polysomnography (NPSG) between April 2018 and June 2023 were included. The SPIN cohort is a comprehensive biomarker platform for the discovery and validation of biomarkers across individuals with neurodegenerative dementias, mild cognitive impairment and CU controls. Recruitment of CU participants followed the established protocol.^54^ Individuals with active psychiatric symptoms or sleep disturbance requiring medication adjustment within the past 5 years, as well as those with neurological or medical conditions or undergoing treatments that could influence brain structure, cognitive function, study adherence or CSF/plasma measures, were excluded.

None of the participants had been previously referred for evaluation of a suspected sleep disorder. Prior to NPSG, participants completed standardized sleep questionnaires, including the validated Spanish version of the Pittsburgh Sleep Quality Index to assess self-reported sleep quality, with scores above 5 considered indicative of poor sleep quality;^55^ the Epworth Sleepiness Scale to evaluate daytime somnolence, with scores above 10 considered indicative of excessive sleepiness;^56^ and the Berlin Questionnaire to screen for risk of OSA.^57^ Questionnaire abnormalities were evaluated by a sleep specialist, who excluded any clinically significant suspicion of sleep disorder. Undiagnosed sleep-breathing disorders were not an exclusion criteria.

All participants underwent a thorough medical history review, physical examination, and standard neuropsychological evaluation, followed by lumbar puncture and blood collection from morning to early afternoon on the same day. Neuropsychological performance was within normal range for age and education, and all participants had a clinical dementia rating of 0, as per the SPIN neuropsychological protocol.^54^ None of the participants met criteria for Alzheimer’s disease biomarker positivity, as determined by established CSF biomarker cutoffs (Aβ42/Aβ40 ratio, pTau181 and tTau) within the past 4 years or by a negative plasma pTau217 result.^58,59^

### Polysomnography

Sleep recordings were conducted during a single night in individual, sound-attenuated, temperature-regulated rooms at the sleep laboratory, supervised by qualified technicians, as described in previous works.^60,61^ Briefly, NPSG included 19 EEG channels according to the international electrode 10–20 system. Data were acquired using the Compumedics E Series System (Compumedics, VIC, Australia) and analysed with Profusion Sleep Software (Compumedics PSG3 version 3.4). Sleep variables were visually scored in 30-s epochs by an accredited technologist, following the guidelines of the American Academy of Sleep Medicine.^62^

Sleep architecture parameters included total sleep time (TST); wake after sleep onset (WASO, in minutes, defined as the time awake after sleep onset); SE (in %, defined as the ratio between total sleep time and time in bed) and SL (in minutes, defined as the time spent awake before the first sleep epoch). Time spent in each stage (N1, N2, N3 and REM) was recorded both in minutes and as a percentage of TST. Sleep fragmentation was assessed using the number of NREM-REM stage transition (stage shift), the total number of cortical arousals throughout the night (arousal total) and the number of awakenings.^62^

Quantitative analysis of SWA was performed by calculating the average power density within the 0.5–4.0 Hz ranges from full-night EEG recording at bilateral frontal, central and parietal electrodes. The mean SWA was then derived by averaging the SWA values across all electrodes.^63^

Respiratory parameters included the apnoea–hypopnea index (AHI), defined as the number of apnoeas (>90% airflow reduction for ≥10 s) and hypopneas (≥30% airflow reduction for ≥10 s with ≥3% oxygen desaturation or cortical arousal) per hour of sleep; the percentage of total sleep time spent with oxygen saturation below 90% (T90) and the presence and severity of OSA, classified according to standard AHI thresholds.^64^

### Blood and CSF collection and analysis

Blood samples were collected in 10 ml ethylenediaminetetraacetic acid tubes. CSF samples were collected on the same day of blood extraction and processed in polypropylene tubes following international recommendations.^65^ All samples were transferred to our laboratory, centrifuged and aliquoted within 2 h of collection and stored at −80°C until analysis. The pre-analytical processing protocol for blood and CSF in the SPIN cohort has been described in detail.^54^

CSF and plasma GFAP concentrations were quantified using commercially available assays on the Single Molecule Array (SIMOA) SR-X platform (Simoa® GFAP Discovery kit; Quanterix, Ref# 102336). Plasma NfL was measured with the Simoa® NF-light Advantage V2 kit (Quanterix, Ref# 104073). CSF YKL-40 concentrations were measured using enzyme-linked immunosorbent assay methods previously described (MicroVue™ YKL-40, Quidel, Ref# 8020).^58,66^ All samples and calibration curves were run in duplicate, with intra- and inter-assay coefficients of variation as reported in prior study.^67^ Core Alzheimer’s disease biomarkers (Aβ42, Aβ40, pTau181 and tTau) in CSF were analysed using the fully automated Lumipulse G600II platform (Fujirebio-Europe), following the manufacturers’ instructions.

### *APOE* status determination

DNA was extracted from peripheral blood and *APOE* genotyping was performed using PCR amplification followed by Sanger sequencing. Participants carrying one or more copies of the ε4 allele were classified as ε4+, whereas the remaining participants were placed in the ε4− group.^68^

### Statistical analyses

Descriptive statistics were reported as median and interquartile range (IQR) for continuous variables and as absolute and relative frequencies for categorical variables. Group comparisons were performed using the Wilcoxon rank-sum test for continuous variables and Fisher’s exact test for categorical variables. Normality was evaluated by visual inspection of the histogram and the Shapiro–Wilk test. Right-skewed variables (CSF GFAP and YKL-40, plasma NfL, CSF pTau181 and tTau, stage shift and SWA) were log-transformed prior to regression analyses to achieve normal distribution. Log transformation also provided the best correction for plasma GFAP, although its distribution remained slightly skewed. CSF Aβ42 and Aβ40 were normally distributed and therefore not transformed.

Associations between sleep parameters, fluid biomarkers and age were examined using Spearman’s rank correlation. Multiple linear regression models were then applied to identify independent sleep-related predictors of biomarker concentrations, adjusting for age, sex, body mass index (BMI), *APOE* ε4 genotype and OSA status, classified as present [apnoea–hypopnea index (AHI) ≥ 5 events/h] or absent (AHI < 5 events/h).

Prior to regression analyses, bivariate correlations among sleep parameters were screened to identify potential redundancy. In case of high correlation (Spearman’s *ρ* > 0.7), the variables were modelled separately in parallel regression models and the model with the highest adjusted *R*^2^ was retained for further analysis.

Predictor selection followed a stepwise forward–backward procedure using the step function from the R base ‘stats’ package. The best-fitting models were identified based on the lowest Akaike information criterion. Multicollinearity was assessed using the variance inflation factor, with values < 5 considered acceptable. Model assumptions were evaluated using standard diagnostic plots, including residual versus fitted values, Q–Q plots, scale-location plot and residuals versus leverage, to assess linearity, homoscedasticity, normality of residuals and influential observations. Potential outliers were identified using an extreme value criterion (3 × IQR) and model diagnostics. Regression models were re-run with and without these values, and results remained unchanged; therefore, all data points were retained in the final analyses.

Moderation analyses were conducted to examine whether age, sex or *APOE* ε4 genotype modified the associations identified between sleep parameters and fluid biomarkers. For each final multiple linear regression model, an interaction term was applied between the relevant sleep predictors and the moderator of interest, while retaining the covariates identified in the main regression models. Model selection and diagnostic checks followed the same procedure as applied in the primary analyses. Moderation was defined as a significant interaction between the sleep parameter and the moderator.

Statistical significance was set at 5% (*P* < 0.05). For Spearman’s correlation, significance was determined based on 95% confidence intervals (CIs) using 5000-sample bootstrapping method, with the exclusion of zero indicating significance (*α* = 0.05). Continuous predictor variables were *z*-scored prior to regression and moderation analyses to allow comparison of effect sizes across measures, while outcome variables were analysed without standardization.

Data cleaning, analysis and visualization were performed using R statistical software (v4.4.1) and the following packages: ‘tidyverse’(v.2.0.0), ‘dplyr’(v.1.1.4), ‘skimr‘(v.2.1.5), ‘rstatix’(v.0.7.2), ‘gtsummary’(v.1.7.3), ‘boot’(v.1.3.31), ‘car’(v3.1.3), ‘ggplot2’(v.3.5.1), ‘patchwork’(v.1.2.0), ‘interactions’(v.1.2.0), ‘emmeans’(v.1.11.2) and the base ‘stats’ package.

## Results

### Participants characteristics

Table 1 summarizes the demographic, cognitive and clinical characteristics of the participants, along with raw biomarker concentrations and sleep parameters. Participants had a median age of 55 years (IQR: 12) and were predominantly female (71%). They were also highly educated, with a median of 17 years of education (IQR: 7). Overall, 25% were *APOE* ε4 carriers, including two individuals homozygous for the ε4 allele. The cohort was generally in good health and was, on average, within the normal weight range [median BMI: 24 (IQR: 4.6)]. Six participants (12%) reported the use of psychotropic medication. Two (4%) were taking antidepressants: one escitalopram and the other venlafaxine. Another two participants (4%) used hypnotics, clonazepam or alprazolam. The remaining two (4%) were on combined treatment with antidepressants and hypnotics: one with escitalopram and lormetazepam, and the other with citalopram and doxylamine. All medications were administered at low doses, and hypnotics were taken on an as-needed basis and were not used the night prior to the NPSG in any of the four cases. No participants were taking anti-seizure or antipsychotic medications. These medications were not expected to influence study outcomes.

**Table 1:** Participant characteristics: demographic, clinical, biomarker and sleep architecture parameters (*N* = 51)

**Sociodemographic and clinical characteristics**		**NPSG sleep architecture parameters**	
Age (years)	55 [12]	Total sleep time (min)	368 [74]
Sex (female)	36 (71%)	Wake after sleep onset (min)	146 [103]
Education (years)	17 [7]	Sleep efficiency (%)	82 [14]
APOEε4+	13 (25%)	Sleep latency (min)	22 [22]
MMSE	30 [1]	Stage N1 (min)/(%)	39 [37]/12 [11]
Hypertension	11 (22%)	Stage N2 (min)/(%)	179 [58]/51 [9]
Dyslipidaemia	13 (25%)	Stage N3 (min)/(%)	73 [30]/19 [8]
Diabetes	2 (3.9%)	Stage REM (min)/(%)	68 [29]/18.2 [6]
Antidepressant treatment	4 (8%)	Number of stage shifts	157 [65]
Hypnotic treatment	4 (8%)	Number of arousal	14 [11]
BMI (kg/m^2^)	24 [4.6]	Number of awakening	21 [8]
Time from NPSG to biofluid collection (days)	1 [8]	Log10 (mean slow wave activity)	225 [180]
**Fluid biomarkers**		**NPSG respiratory parameters**	
CSF YKL-40 (pg/ml)	184 [87]	OSA (AHI > 5)	15 (29%)
CSF GFAP (pg/ml)	6389 [3795]	Moderate/severe OSA (AHI > 15)	5 (9.8%)
Plasma GFAP (pg/ml)	105 [92]	AHI (events/h)	3 [5]
Plasma NfL (pg/ml)	7.8 [4.7]	T90 (%)	0.1 [0.5]
CSF Aβ40	11 063 [3543]	**Sleep questionnaire scores**	
CSF Aβ42	1135 [501]	PSQI	4 [3]
CSF pTau181 (pg/ml)	33 [12]	ESS	4 [6]
CSF tTau (pg/ml)	244 [96]	BQ (high risk)	3 (6%)

Values are presented as median (IQR) or *n* (%).

*APOE*ε4+, carrier of one or two *APOE* ε4 alleles, MMSE, Mini-Mental state examination; Aβ40, amyloid-beta 1–40; Aβ42, amyloid-beta 1–42; pTau181, Tau protein phosphorylated at threonine 181; tTau, total tau; NPSG, nocturnal polysomnography; T90, time proportion with oxygen saturation below 90% during sleep; PSQI, Pittsburgh Sleep Quality Index; ESS, Epworth Sleepiness Scale; BQ, Berlin Questionnaire.

All mean sleep questionnaire parameters were within the normal range, with only three participants classified as being at risk for OSA. All participants underwent NPSG and had plasma available for biomarker determination. The median interval between NPSG and sample collection was 1 day (IQR: 8; range: 1–127), with 33 participants (65%) undergoing NPSG the night before medical evaluation and sample collection. CSF samples were available for 47 individuals (92.2%), none of whom met criteria for Alzheimer’s disease biomarker positivity. Of the four participants without available CSF, three had previously shown normal CSF biomarker profiles in samples obtained within the past 4 years and one had a negative plasma pTau217 result.

Sleep parameters were within normal ranges, with mildly reduced sleep efficiency (>85% considered normal). The median AHI was within normal limits (<5/h). Fifteen participants had OSA, including one with moderate OSA (AHI ≥ 15) and four with severe OSA (AHI ≥ 30).

Age was positively associated with CSF YKL-40, CSF GFAP, plasma GFAP and plasma NfL concentrations (*ρ* = 0.62, 0.47, 0.47 and 0.66, respectively, all *P* < 0.05) (Supplementary Table 1), as well as with N1 stage duration, stage shift, arousal total, AHI and T90 (*ρ* = 0.34–0.44; all *P* < 0.05) (Supplementary Table 1). In contrast, it was negatively associated with SWA (*r* = −0.39, *P* < 0.05) (Supplementary Table 1). Associations among the fluid biomarkers are reported in Supplementary Table 2.

Compared to females, males had a higher median age, and the proportion of individuals with hypertension was greater. Males also showed reduced TST and SE and increased respiratory events, although the proportion of moderate-to-severe OSA cases did not differ significantly between genders. CSF YKL-40 concentrations were higher in males compared to females (Supplementary Table 3).

No significant associations were found between BMI and sleep parameters or biomarker concentrations. Additionally, no significant demographic, clinical, biomarker or sleep parameter differences were observed between participants who had fluid collection the morning after NPSG and those who completed NPSG on a separate day. Similarly, biomarker concentrations did not differ between participants with moderate-to-severe OSA and those without.

### Bivariate associations between sleep parameters and fluid biomarkers

**Figure 1:**
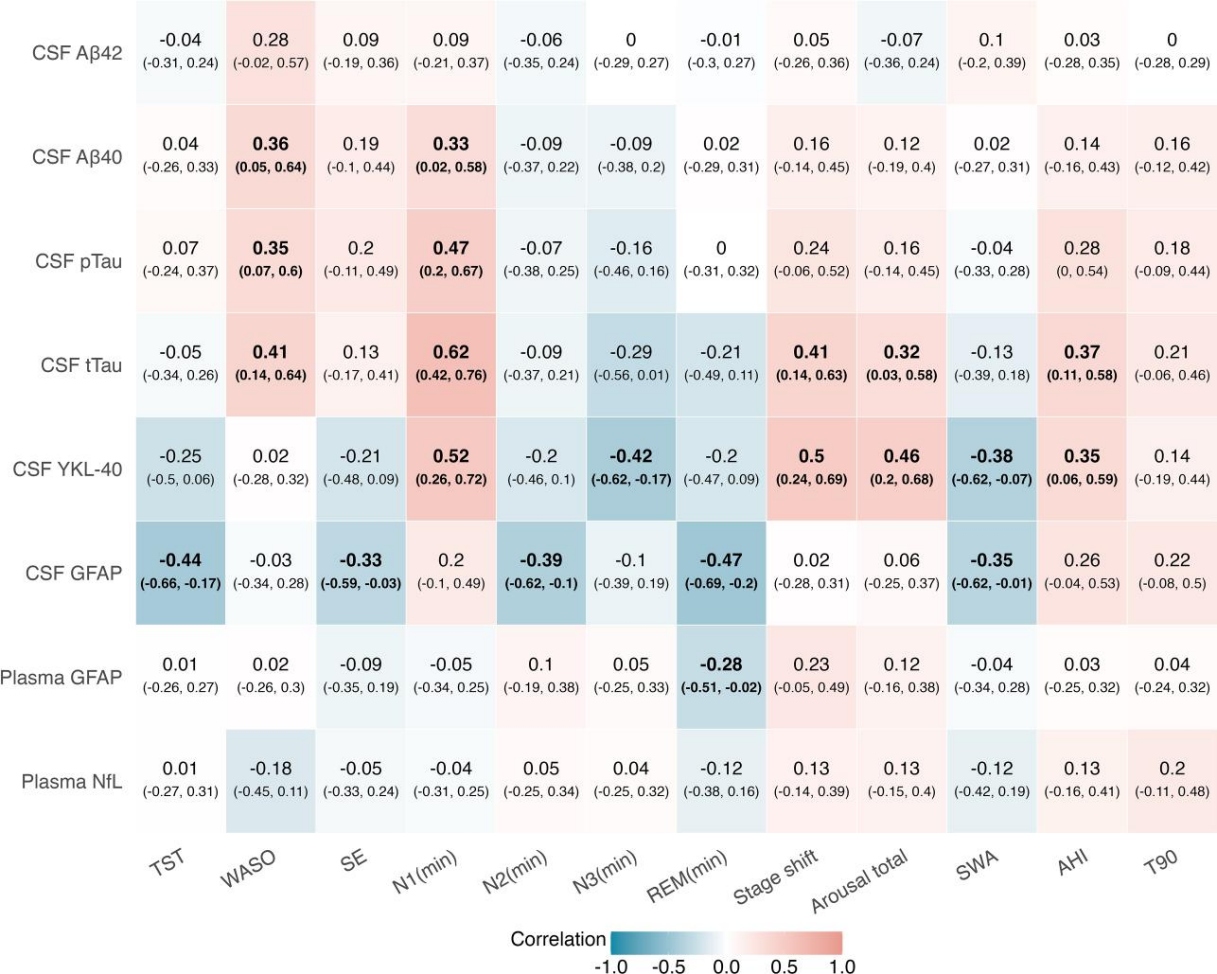
**Spearman’s correlation coefficients between objective sleep parameters and fluid biomarker concentrations.** Correlation coefficient was computed between sleep parameters including macroarchitecture (TST, WASO, SE, N1, N2, N3 and REM stage duration), sleep fragmentation (stage shift, arousal total), microstructure (SWA) and respiratory parameters (AHI, T90) and Alzheimer’s disease-related biomarkers, including CSF YKL-40, GFAP, Aβ40, Aβ42, pTau181, tTau, plasma GFAP and NfL. Values represent Spearman’s *ρ* and 95% CIs (in parentheses), computed via bootstrapping (5000 samples) (*N* = 47 for correlations with CSF biomarkers; *N* = 51 for correlations with plasma biomarkers). log, logarithmic transformation;Aβ, amyloid-β; pTau, phosphorylated tau (Thr181); tTau, total tau; N1(min), N1 stage duration; N2(min), N2 stage duration; N3(min), N3 stage duration; REM(min), REM stage duration; stage shift, number of stage shifts; arousal total, total number of arousals; T90, percentage of total sleep time with oxygen saturation <90%.

A Spearman correlation analysis (Fig. 1) revealed that more superficial and fragmented sleep, reflected by longer N1 duration, higher WASO, more transition between sleep stages (stage shift) and a greater number of arousals, was associated with higher concentrations of CSF YKL-40 (*ρ* = 0.52, 0.46 and 0.50 for N1, stage shift and arousals, respectively), as well as with elevated concentrations of CSF Aβ40 (*ρ* = 0.33 with N1 and *ρ* = 0.36 with WASO), CSF pTau181 (*ρ* = 0.47 with N1 and *ρ* = 0.35 with WASO) and CSF tTau (*ρ* = 0.62, 0.41, 0.41 and 0.31 for N1, WASO, stage shift and arousals, respectively).

In contrast, deeper sleep parameters, including longer N3 duration and increased SWA, were associated with lower CSF YKL-40 (*ρ* = −0.42 and −0.38, respectively) and CSF GFAP (*ρ* = −0.35 with SWA) concentrations. Additionally, longer TST, higher SE and longer N2 and REM durations were negatively associated with CSF GFAP (*ρ* = −0.44, −0.33, −0.39 and −0.47, respectively).

Regarding respiratory parameters, AHI was positively associated with CSF YKL-40 (*ρ* = 0.35) and CSF tTau (*ρ* = 0.37) concentrations, while T90 showed no significant associations with the biomarkers evaluated.

Overall, no relevant associations were observed between sleep parameters and plasma biomarkers, nor with CSF Aβ42.

### Multivariate associations between sleep parameters and astrocytic-neuroaxonal fluid biomarkers

**Figure 2:**
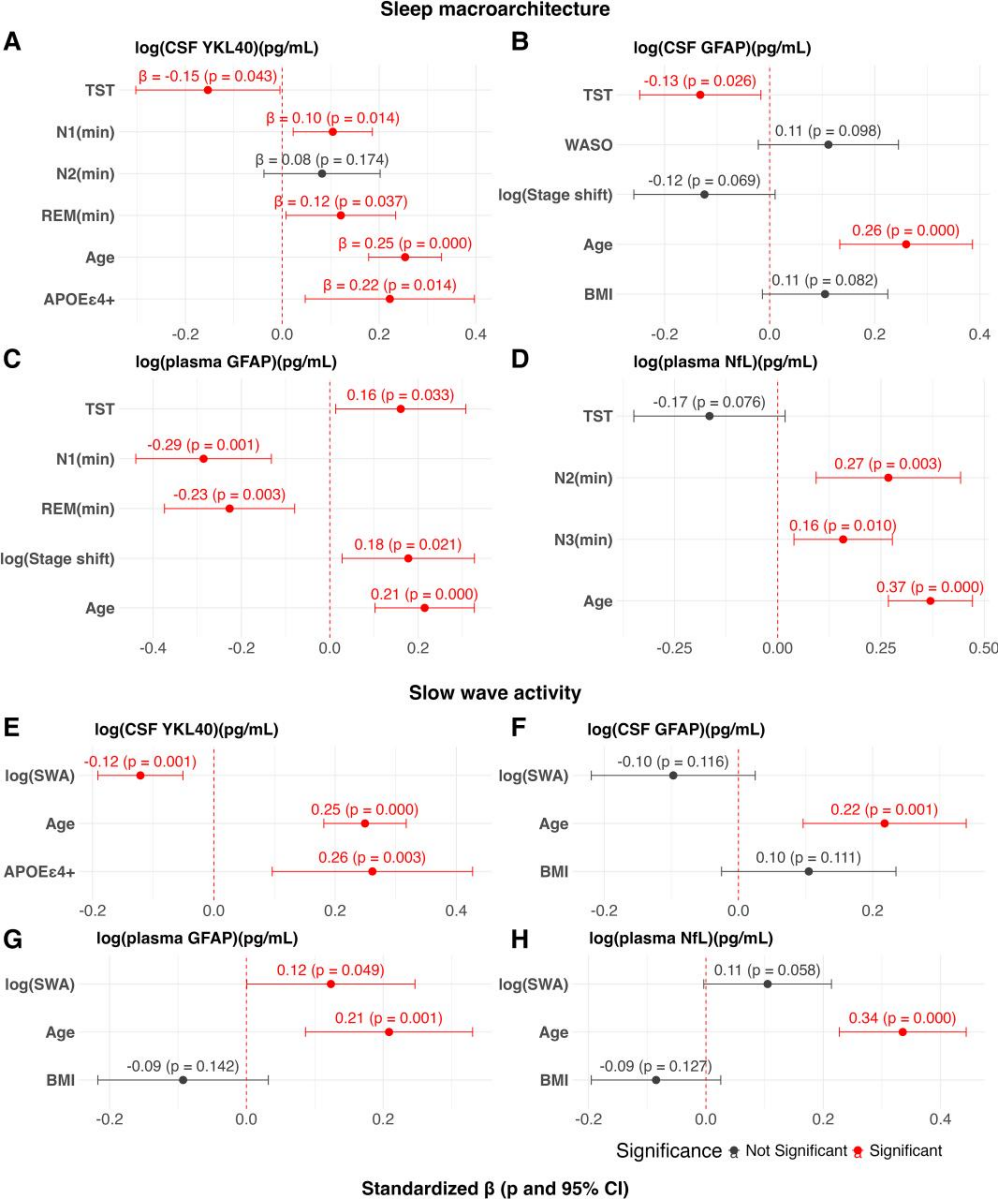
**Regression models for sleep architecture parameters and astrocytic-neuroaxonal fluid biomarker concentrations. (A–H)** Standardized regression coefficients (dots indicating the estimated *β*), 95% CIs (bars) and corresponding *P*-values for predictors retained in the final model after stepwise variable selection (both forward and backward). Models were adjusted for age, sex, BMI, *APOE* ε4 genotype and OSA where appropriate. Associations reaching statistical significance (*P* < 0.05 and 95% CI not including zero) are indicated accordingly. Biomarkers and skewed sleep parameters (stage shift and SWA) were log-transformed prior to analysis when necessary to meet normality assumptions (*N* = 47 for models with CSF biomarkers; *N* = 51 for models with plasma biomarkers). log, logarithmic transformation; N1(min), N1 stage duration; N2(min), N2 stage duration; N3(min), N3 stage duration; REM(min), REM stage duration; stage shift, number of stage shifts; APOE ε4+, APOE ε4 carrier.

Multiple linear regression models used to identify independent sleep architecture predictors of astrocytic and neuroaxonal biomarker concentrations are presented in Fig. 2 and summarized in Table 2.

**Table 2:** Summary of multiple linear regression models to predict astrocytic-neuroaxonal fluid biomarkers

	RSE	Adjusted *R*^2^	*F*-statistic	*P*-value
For sleep architecture parameters				
log(CSF YKL-40)	0.235	0.6139	13.19	<0.0001
log(CSF GFAP)	0.3768	0.3744	6.505	<0.0001
log(Plasma GFAP)	0.3619	0.3946	7.518	<0.0001
log(Plasma NfL)	0.3424	0.5157	14.31	<0.0001
For slow wave activity				
log(CSF YKL-40)	0.2282	0.6434	27.46	<0.0001
log(CSF GFAP)	0.4085	0.2962	7.173	0.0006
log(Plasma GFAP)	0.414	0.2076	5.192	0.004
log(Plasma NfL)	0.3662	0.445	13.83	<0.0001

In the model for CSF YKL-40 (adjusted *R*^2^ = 0.61, *P* < 0.0001), longer N1 and REM duration were positively associated with CSF YKL-40 concentrations (*β* = 0.10, *P* = 0.014; *β* = 0.12, *P* = 0.037, respectively), while TST showed a negative association (*β* = −0.15, *P* = 0.043). Age and *APOE* ε4 genotype were also significant predictors of CSF YKL-40 concentrations (*β* = 0.25, *P* < 0.001; *β* = 0.22, *P* = 0.014, respectively) (Fig. 2A).

For CSF GFAP (adjusted *R*^2^ = 0.37, *P* < 0.0001), TST was the only sleep-related parameter that remained significant (*β* = −0.13, *P* = 0.026). Age was also associated with biomarker concentrations (*β* = 0.26, *P* < 0.001), while WASO, stage shift and BMI did not reach significance (Fig. 2B).

For plasma GFAP (adjusted *R*^2^ = 0.39, *P* < 0.0001), longer N1 and REM duration were negatively associated with biomarker concentration (*β* = −0.29, *P* = 0.001; *β* = −0.23, *P* = 0.003, respectively), whereas TST and stage shift were positively associated (*β* = 0.16, *P* = 0.033; *β* = 0.18, *P* = 0.021, respectively). Age again remained a significant predictor (*β* = 0.21, *P* < 0.001) (Fig. 2C).

In the model for plasma NfL (adjusted *R*^2^ = 0.52, *P* < 0.0001), longer N2 and N3 duration were independently associated with higher concentrations (*β* = 0.27, *P* = 0.003; *β* = 0.16, *P* = 0.01). Age showed a strong positive association (*β* = 0.37, *P* < 0.001) (Fig. 2D).

In models evaluating SWA separately, higher SWA was independently associated with lower concentrations of CSF YKL-40 (*β* = −0.12, *P* = 0.001) and higher concentrations of plasma GFAP (*β* = 0.12, *P* = 0.049) (Fig. 2E and G). No significant associations were found for CSF GFAP or plasma NfL (Fig. 2F and H). Among all models, the one predicting CSF YKL-40 showed the highest explanatory power (adjusted *R*^2^ = 0.6139) (Table 2). Notably, models using SWA measured at different electrode locations (Fz, Cz and Pz) yielded similar results, with no significant differences in model performance across derivations.

### Multivariate associations between sleep parameters and CSF Alzheimer’s disease-related proteins

**Figure 3:**
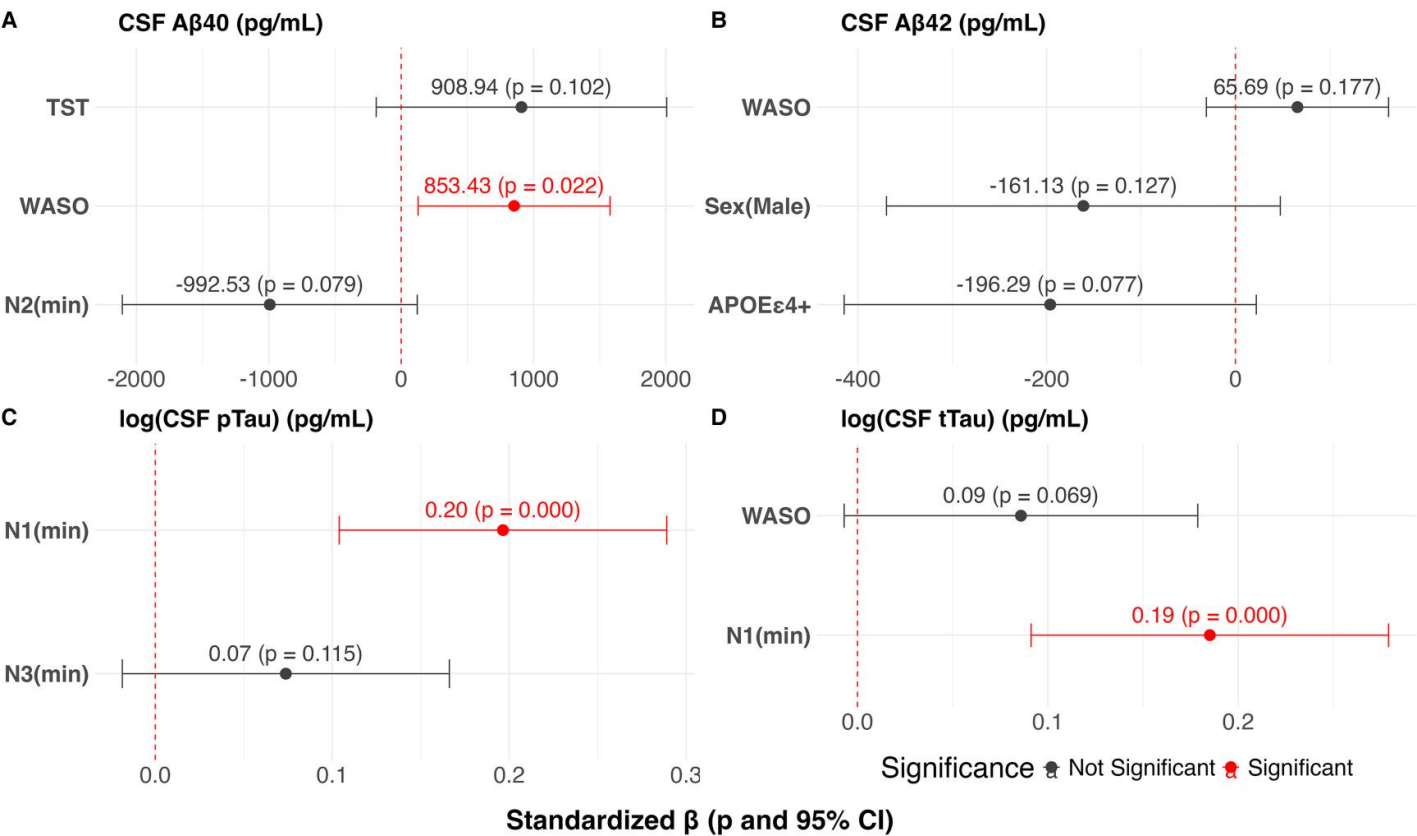
**Regression models for sleep architecture parameters and CSF core Alzheimer’s disease-related proteins. (A–D)** Standardized regression coefficients (dots indicating the estimated *β*), 95% CIs (bars) and corresponding *P*-values for predictors retained in the final model after stepwise variable selection (both forward and backward). Models were adjusted for age, sex, BMI, *APOE* ε4 genotype and OSA where appropriate. Associations reaching statistical significance (*P* < 0.05 and 95% CI not including zero) are indicated accordingly (*N* = 47 for models with CSF biomarkers). log, logarithmic transformationpTau, phosphorylated tau (Thr181); tTau, total tau; N1(min), N1 stage duration; N2(min), N2 stage duration; N3(min), N3 stage duration; APOE ε4+, APOE ε4 carrier.

For CSF Aβ40 and CSF Aβ42, the models were statistically significant (*P* = 0.047 and 0.042, respectively) but explained only a small proportion of variance (adjusted *R*^2^ = 0.11 for both). WASO was the only sleep parameter independently associated with Aβ40 (*β* = 853.43, *P* = 0.022), while no significant associations were found for CSF Aβ42 (Fig. 3A and B).

Regarding CSF pTau181 and CSF tTau, longer N1 duration was independently associated with higher biomarkers concentrations (*β* = 0.20, *P* < 0.001; *β* = 0.19, *P* < 0.001, respectively), although models fit remained modest (adjusted *R*^2^ = 0.27 and 0.36, respectively) (Fig. 3C and D).

SWA was not associated with any of the core Alzheimer’s disease-related proteins, and the corresponding regression models did not reach statistical significance (Table 3).

**Table 3:** Summary of multiple linear regression models to predict CSF Alzheimer’s disease-related proteins

	RSE	Adjusted *R*^2^	*F*-statistic	*P*-value
For sleep architecture parameters				
CSF AB40	2431	0.1092	2.879	0.047
CSF AB42	314	0.1146	2.984	0.042
log(CSF pTau181)	0.258	0.2704	9.523	0.0004
log(CSF tTau)	0.291	0.3642	14.18	<0.0001
For slow wave activity				
CSF AB40	2452	0.0226	1.51	0.2327
CSF AB42	647.57	0.0726	2.722	0.0773
log(CSF tTau)	0.366	0.0262	2.184	0.1468

### Moderating effects of *APOE* ε4, age and sex on the associations between sleep parameters and fluid biomarkers

**Figure 4:**
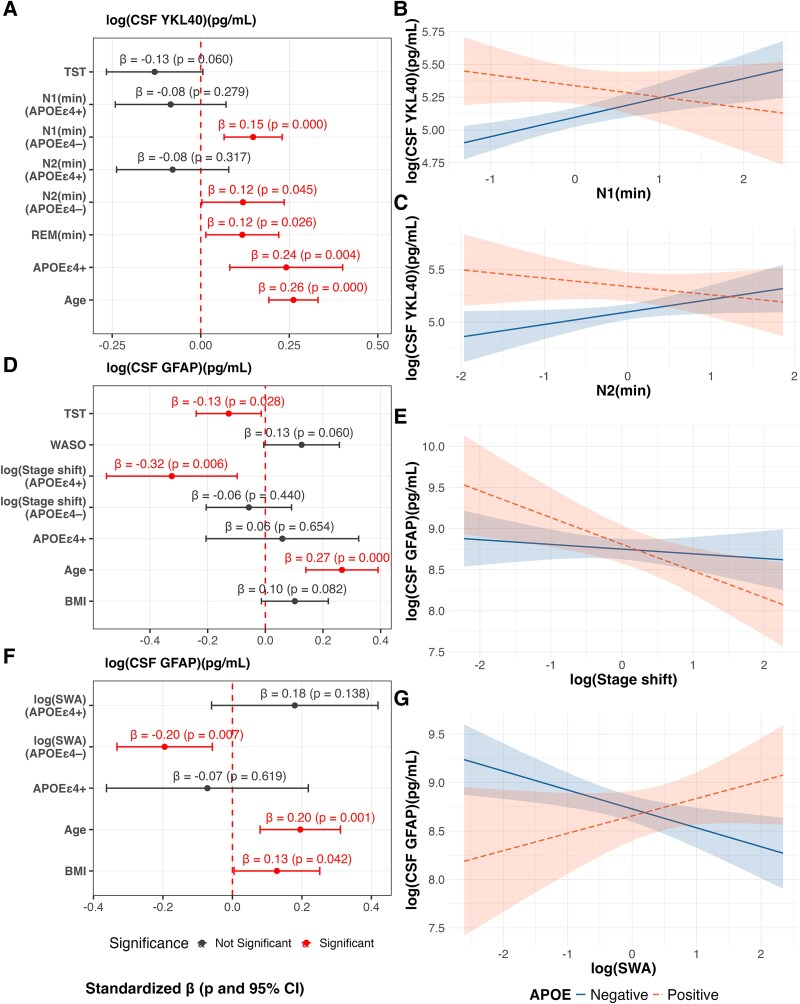
**Moderating effect of *APOE* ε4 genotype on the association between sleep parameters and fluid biomarkers (left panels: A, D, F).** Forest plots showing standardized regression coefficients (dots indicating *β*), 95% CIs (bars) and *P*-values from multivariate models including interaction terms between *APOE* ε4 genotype and sleep parameters, adjusted for age, sex, BMI and OSA where appropriate. Statistically significant effects (*P* < 0.05 and 95% CI not including zero) are shown in red. Biomarkers and skewed sleep parameters (e.g. stage shift, SWA) were log-transformed prior to analysis (*N* = 47 for models with CSF biomarkers; *N* = 51 for models with plasma biomarkers) (right panels: **B, C, E, G**). Interaction plots depicting predicted associations stratified by *APOE* ε4 carrier status (non-carriers in blue, carriers in pink), adjusted for covariates where appropriate. Shaded areas represent 95% CIs. Biomarkers and skewed sleep parameters (e.g. stage shift, SWA) were log-transformed prior to analysis (*N* = 47 for models with CSF biomarkers; *N* = 51 for models with plasma biomarkers). log, logarithmic transformation; N1(min), N1 stage duration; N2(min), N2 stage duration; REM(min), REM stage duration; stage shift, number of stage shifts; APOE ε4+, APOE ε4 carrier.

We next examined whether the *APOE* ε4 genotype moderated the associations between sleep parameters and fluid biomarkers. Significant interactions were observed for CSF YKL-40 and GFAP (Fig. 4; Table 4). For CSF YKL-40, *APOE* ε4 significantly moderated the relationship with N1 and N2 duration (interaction terms: *β* = −0.23, *P* = 0.009; *β* = −0.20, *P* = 0.012, respectively). Stratified analyses indicated that longer N1 and N2 durations were positively associated with higher CSF YKL-40 concentrations in non-carriers (*β* = 0.15, *P* < 0.001; *β* = 0.12, *P* = 0.045, respectively) but not in ε4 carriers (Fig. 4A–C). For CSF GFAP, a significant interaction was detected between stage shift and *APOE* ε4 carriers (interaction term: *β* = −0.27, *P* = 0.035), with a negative association observed in carriers (*β* = −0.32, *P* = 0.006), whereas this relationship was attenuated and not statistically significant in non-carriers (Fig. 4D and E). In the SWA-specific model, *APOE* ε4 also moderated the associations with CSF GFAP (interaction term: *β* = 0.37, *P* = 0.010). Higher SWA was associated with lower CSF GFAP concentrations in non-carriers (*β* = −0.20, *P* = 0.007), while no significant association was observed in ε4 carriers (Fig. 4F and G).

**Table 4:** Summary of moderation models by *APOE* ε4 genotype

	RSE	Adjusted *R*^2^	*F*-statistic	*P*-value
For sleep architecture parameters				
log(CSF YKL-40)	0.2131	0.6824	13.35	<0.0001
log(CSF GFAP)	0.3647	0.4141	5.645	0.0002
For slow wave activity				
log(CSF GFAP)	0.3836	0.3795	6.382	0.0002

Regarding the moderating role of age, a significant interaction was found for SWA and age in predicting plasma GFAP concentration (interaction term: *β* = 0.15, *P* = 0.021). Stratified analyses indicated that higher SWA was associated with increased plasma GFAP in older participants (+1 SD of age) (*β* = 0.21, *P* = 0.004), whereas no significant association was observed in younger individuals (−1 SD of age) (Supplementary Fig. 1A and B; Supplementary Table 4).

Regarding sex, significant interactions were observed for N1 and REM duration on CSF YKL-40 (interaction terms: *β* = −0.17, *P* = 0.018; *β* = −0.24, *P* = 0.003, respectively). These associations were positive in females (*β* = 0.19, *P* < 0.001; *β* = 0.19, *P* = 0.003, respectively), while they were attenuated or absent in males (Supplementary Fig. 1C–E). Model fit remained high when this interaction was considered (adjusted *R*^2^ = 0.68, *P* < 0.0001; Supplementary Table 4).

No moderating effects of these factors were observed on Alzheimer’s disease-related protein concentrations.

## Discussion

In this study, we found that specific features of sleep architecture were related to CSF concentrations of astrocytic biomarkers. Lighter and more fragmented sleep was linked to higher CSF YKL-40, whereas deeper and more consolidated sleep was associated with lower concentrations of CSF GFAP. Interestingly, plasma GFAP showed an inverse profile. We also observed significant associations between REM sleep and astrocytic biofluid markers. In addition, age, sex and *APOE* ε4 genotype significantly influenced several of these associations.

These findings are consistent with existing evidence linking sleep disruption to increased neuroinflammatory activity. Both experimental and observational studies have shown that disrupted sleep can trigger systemic and central immune responses via proinflammatory mediators.^25^ In our study, lighter and more fragmented sleep was associated with elevated CSF YKL-40, whereas deeper and more consolidated sleep was linked to lower concentrations of astrocytic fluid biomarkers. These findings support previous reports of increased glial activation in individuals with chronic insomnia or reduced deep sleep.^16,30-33,49^ Similarly, in older adults, sleep fragmentation has been associated with upregulation of astrocyte-related genes, while animal models demonstrate that sleep loss induces astrocytic phagocytosis and microglial activation.^69,70^ In parallel, superficial sleep has also been associated with higher concentrations of Alzheimer’s disease-related proteins. In our sample, lighter sleep predicted higher concentrations of CSF Aβ40, pTau181 and tTau, supporting prior evidence that experimental sleep deprivation elevates amyloid-β and tau in CU individuals.^5,6,71^ Altogether, these results suggest that both astrocytic and Alzheimer’s disease-related biomarkers are associated with subtle variations in sleep physiology.

Interestingly, plasma biomarkers displayed an opposite profile to CSF markers. Plasma GFAP increased with longer TST and higher SWA, and decreased with longer N1 and REM duration. Likewise, plasma NfL was positively associated with N3 duration and showed a trend with SWA. These patterns may reflect enhanced activity of the glymphatic system, a brain-wide clearance mechanism that facilitates the removal of metabolic waste from the interstitial space via perivascular pathway and is most active during deep non-REM sleep.^72,73^ Astrocytes contribute critically to this process by regulating fluids dynamics through their perivascular endfeet and maintaining the environment needed for solute transport.^23,24^ While most evidence comes from animal models, our findings support the idea that deep sleep promotes astrocyte-mediated clearance even in CU individuals. The concurrent increase in plasma GFAP and NfL further suggests that restorative sleep may enhance the redistribution of central nervous system proteins into the periphery, potentially contributing to the limited correlation sometimes observed between central and peripheral biomarker measures.^74^ A small cross-over study with intensive sampling similarly found that sleep deprivation increased CSF but decreased plasma Aβ and tau concentrations, supporting the notion of differential biomarker dynamics across compartments, albeit requiring confirmation in larger cohorts.^75^

In contrast, we observed no meaningful link between SWA and CSF Alzheimer’s disease-related proteins. This diverges from previous studies showing increased tau and amyloid-β in relation to reduced SWA.^6-8^ Several methodological differences may account for this discrepancy. Prior studies often included older participants with an average age at least 10 years older than in our sample, and in some cases, more than 50% were amyloid-positive based on PET or CSF biomarkers.^7,8^ Additionally, some employed sleep deprivation protocols, which may amplify sleep–biomarker relationship beyond physiological conditions.^6^ In contrast, our cohort included younger, amyloid-negative individuals in whom glymphatic clearance and other compensatory mechanisms are expected to be intact, potentially limiting the detectability of associations between SWA and Alzheimer’s disease-related proteins.

In addition to the associations observed with NREM sleep, REM sleep duration was also significantly related to astrocytic fluid biomarkers. The mechanisms by which astrocytic activity interacts with REM sleep regulation are not yet fully understood, and further work specifically addressing this relationship is needed. Among the few studies available, animal models showed that pontine astrocytic activation suppressed REM initiation and reduced REM episodes,^76^ while in Parkinson’s disease, patients with REM sleep behaviour disorders (RBD) exhibited higher plasma GFAP and NfL concentrations than those without RBD.^77^ Similarly, individuals with isolated RBD (iRBD) presented with elevated plasma GFAP and NfL in the absence of increased pTau181.^78^ Together, these findings pointed to a relationship between REM sleep and astroglial activity, and our result suggested that such associations were also present in CU individuals without biomarker evidence of Alzheimer’s disease.

To further understand the complexity of sleep–biomarker interactions, we examined the role of key susceptibility factors. APOE is a key protein involved in lipid transport and metabolism, mainly synthesized by astrocytes in the central nervous system.^43,72^ The ε4 isoform has been linked to altered BBB properties and reduced amyloid-β clearance, including disrupted endothelial junctions and reduced astrocytic end-foot coverage in animal models.^42,45,79^ In CU adults, *APOE* ε4 carriers show poorer sleep quality, characterized by shorter TST and SE and greater WASO and awakenings, compared to non-carriers.^38,80^ Self-reported data further support this pattern.^37^ Our finding expands this evidence by showing that APOE ε4 also moderates the relationship between sleep and astrocytic biomarkers. Specifically, in ε4 carriers, the positive association between N1 and N2 durations and CSF YKL-40 seen on non-carriers were no longer evident. This may reflect genotype-dependent difference in astrocytic activity to sleep features, potentially indicating early glial alterations in individuals genetically predisposed to Alzheimer’s disease. Consistent with this interpretation, post-mortem studies have shown greater sleep impairment in ε4 carriers during the last year of life, even when adjusting for Alzheimer’s disease neuropathology. These findings suggest that APOE ε4 may also influence sleep regulation through additional biological mechanisms, not directly related to Alzheimer’s disease core proteinopathy.^81^ Whether these differences in astrocytic activity contribute to increased Alzheimer’s disease vulnerability remains to be clarified.

Finally, both age and sex are known to influence sleep physiology and glial responses.^46,48,82-84^ In our study, these factors also emerged as relevant moderators. The association between SWA and plasma GFAP was stronger in older participants, suggesting that the influence of deep sleep on peripheral astrocytic biomarkers may become more prominent with age. Additionally, the associations between CSF YKL-40 and both N1 and REM duration differed by sex, with stronger associations observed in females. These findings highlight the importance of considering individual variability in sleep–astrocyte interactions.

### Strengths and limitations

The strengths of this study include the rigorous selection of cognitively unimpaired participants and the use of gold-standard NPSG alongside comprehensive biomarker analysis in paired CSF and plasma samples. Notably, the short interval between NPSG and biofluid collection, averaging 13.2 days and 65% with biofluid collection the morning after NPSG, is much shorter than in previous studies,^7,49^ where it ranged from several months to years. This is especially relevant for dynamic biomarkers like the Aβ42 and Aβ40, which may be sensitive to timing.

A limitation of this study is the relatively small sample size. However, this size is similar to that in other studies,^7,38,49,80^ and has provided enough statistical power to find interesting associations. In addition, the cross-sectional design precludes any causal inference; longitudinal studies are needed to determine the temporal dynamics and potential predictive value of sleep–biomarker relationship.

The sample was relatively homogeneous, comprising predominantly Caucasian (98%), highly educated individuals without overweight and with low prevalence of medical comorbidities, including sleep-breathing disturbances. This profile enabled the exploration of sleep–biomarker associations in a physiologically healthy context, minimizing potential confounding factors. However, it may limit the generalizability of our findings to more diverse or clinically complex population. In addition, the use of laboratory-based NPSG may also limit ecological validity due to potential alterations in sleep patterns in the artificial environment. Finally, given the practical constraints of clinical procedures, CSF collection was obtained within a 4.5-h interval in over 90% of cases, although not always at the same time of day. This may introduce variability in biomarkers such as Aβ peptides. Which are known to follow a diurnal pattern.

## Conclusion

Our results support the hypothesis that astrocytic biomarkers are associated with physiological sleep variations and reinforce the idea that astrocytic dysregulation may serve as a mechanistic link between sleep disruption, neuroinflammation and neurodegeneration. Moreover, the compartment-specific behaviour of astrocytic fluid biomarkers observed in our study suggests that sleep may contribute to their redistribution from the central to the peripheral compartment. Finally, the modulation of these associations by age, sex and *APOE* ε4 genotype indicates that at-risk individuals may exhibit different patterns in the relationship between sleep and astrocytic fluid biomarkers, even in the absence of overt pathology, potentially contributing to long-term vulnerability.

Given the potential role of sleep as a modifiable factor, future studies should explore how astrocytic biomarkers interact with sleep parameters across the neurodegenerative disease continuum and whether alterations in this function could serve as early indicators or therapeutic targets.

## Supplementary Material

fcaf437_Supplementary_Data

## Data Availability

Raw anonymized data are available upon reasonable request. The code for statistical analysis is provided in Supplementary File 2. All requests for data access should be sent to the corresponding author, including a detailed description of the study hypothesis and statistical analysis plan. The steering committee of this study will decide whether data sharing is appropriate based on the novelty and scientific rigour of the proposal. All applicants will be asked to sign a data access agreement.
