# Supplementary material for: Sleep-related changes in astrocytic biomarkers are modulated by APOE ε4 genotype in cognitively unimpaired adults
Source: Brain Commun. 2025 Nov 7;7(6):fcaf437. doi: 10.1093/braincomms/fcaf437 (PMC12629230; doi:10.1093/braincomms/fcaf437)
Supplement: fcaf437_Supplementary_Data [file fcaf437_supplementary_data.zip › Supplementary_Material_Tables_and_Figure.pdf]

**Supplementary Table 1. Associations of age with NPSG sleep parameters and biofluid biomarkers (Spearman's rho, 95%CI)**					
**Biofluid markers**					
**CSF Aβ40**	**CSF Aβ42**	**CSF pTau**	**CSF tTau**	**CSF YKL-40**	**CSF GFAP**
-0.03<br>[-0.30, 0.25]	-0.16<br>[-0.39, 0.10]	0.14<br>[-0.16, 0.43]	0.25<br>[-0.04, 0.49]	**0.62**<br>**[0.37, 0.79]**	**0.47**<br>**[0.18, 0.69]**
**Plasma GFAP**	**Plasma NfL**				
**0.47**<br>**[0.23, 0.66]**	**0.66**<br>**[0.47, 0.79]**				
**NPSG-related parameters**					
**TST**	**WASO**	**SE**	**Stage N1 (min)**	**Stage N2 (min)**	**Stage N3 (min)**
-0.26<br>[-0.52, 0.027]	-0.12<br>[-0.41, 0.19]	-0.26<br>[-0.53, 0.04]	**0.34**<br>**[0.07, 0.57]**	-0.24<br>[-0.50, 0.05]	-0.15<br>[-0.43, 0.14]
**Stage REM (min)**	**Number of stage shifts**	**TNA**	**SWA**	**AHI**	**T90**
-0.26<br>[-0.50, 0.01]	**0.35**<br>**[0.10, 0.56]**	**0.33**<br>**[0.04, 0.57]**	**-0.39**<br>**[-0.62, -0.12]**	**0.44**<br>**[0.17, 0.66]**	**0.34**<br>**[0.05, 0.60]**

Note: Values represent Spearman's correlation coefficients (rho) with 95% confidence intervals obtained by bootstrap resampling (5,000 iterations). Correlations were considered statistically significant when the 95% confidence interval excluded 0, and significant correlations are shown in bold. Abbreviations. Abbreviations: CSF, cerebrospinal fluid; YKL-40, chitinase-3-like protein 1; GFAP, glial fibrillary acidic protein; NfL, neurofilament light chain; Aβ40, amyloid-β 1-40; Aβ42, amyloid-β 1-42; pTau181, tau protein phosphorylated at threonine 181; tTau, total tau; NPSG, nocturnal polysomnography; TST, total sleep time; WASO, wake after sleep onset; SE, sleep efficiency; TNA, total number of arousals, stage shift, number of transitions between sleep stages; SWA, average slow wave activity; AHI, apnea-hypopnea index; T90, time proportion with oxygen saturation below 90% during sleep; OSA, obstructive sleep apnea; PSQI, Pittsburgh Sleep Quality Index; ESS, Epworth Sleepiness Scale; BQ, Berlin Questionnaire.

Supplementary table 2. Correlations between biofluid biomarkers (Spearman's rho, 95% CI)

	CSF Aβ40	CSF Aβ42	CSF pTau	CSF tTau	CSF YKL-40	CSF GFAP	Plasma GFAP	Plasma NfL
**CSF Aβ40**	1.00	**0.79 [0.57, 0.93]**	**0.88 [0.77, 0.94]**	**0.69 [0.45, 0.85]**	0.3 [0.02, 0.53]	0.11 [-0.19, 0.39]	-0.2 [-0.46, 0.11]	-0.17 [-0.42, 0.12]
**CSF Aβ42**	**0.79 [0.57, 0.93]**	1.00	**0.66 [0.44, 0.82]**	**0.45 [0.18, 0.68]**	0.02 [-0.27, 0.31]	0.16 [-0.13, 0.45]	-0.17 [-0.44, 0.12]	-0.23 [-0.48, 0.04]
**CSF pTau**	**0.88 [0.77, 0.94]**	**0.66 [0.44, 0.82]**	1.00	**0.77 [0.55, 0.92]**	**0.4 [0.14, 0.62]**	0.19 [-0.12, 0.47]	-0.12 [-0.41, 0.19]	-0.04 [-0.3, 0.24]
**CSF tTau**	**0.69 [0.45, 0.85]**	**0.45 [0.18, 0.68]**	**0.77 [0.55, 0.92]**	1.00	**0.54 [0.33, 0.68]**	0.12 [-0.2, 0.4]	0.02 [-0.28, 0.31]	0.05 [-0.21, 0.31]
**CSF YKL-40**	**0.3 [0.02, 0.53]**	0.02 [-0.27, 0.31]	**0.4 [0.14, 0.62]**	**0.54 [0.33, 0.68]**	1.00	0.27 [-0.03,0.53]	0.26 [-0.06, 0.52]	**0.34 [0.05, 0.58]**
**CSF GFAP**	0.11 [-0.19, 0.39]	0.16 [-0.13, 0.45]	0.19 [-0.12, 0.47]	0.12 [-0.2, 0.4]	0.27 [-0.03, 0.53]	1.00	**0.32 [0.03, 0.56]**	0.22 [-0.06, 0.46]
**Plasma GFAP**	-0.2 [-0.46, 0.11]	-0.17 [-0.44, 0.12]	-0.12 [-0.41, 0.19]	0.02 [-0.28, 0.31]	0.26 [-0.06, 0.52]	**0.32 [0.03, 0.56]**	1.00	**0.71 [0.48, 0.85]**
**Plasma NfL**	-0.17 [-0.42, 0.12]	-0.23 [-0.48, 0.04]	-0.04 [-0.3, 0.24]	0.05 [-0.21, 0.31]	**0.34 [0.05, 0.58]**	0.22 [-0.06, 0.46]	**0.71 [0.48, 0.85]**	1.00

Note: Values represent Spearman's correlation coefficients (rho) with 95% confidence intervals obtained by bootstrap resampling (5,000 iterations). Correlations were considered statistically significant when the 95% confidence interval excluded , and significant correlations are shown in bold. Abbreviations: CSF, cerebrospinal fluid; YKL-40, chitinase-3-like protein 1; GFAP, glial fibrillary acidic protein; NfL, neurofilament light chain; Aβ40, amyloid-β 1-40; Aβ42, amyloid-β 1-42; pTau181, tau protein phosphorylated at threonine 181; tTau, total tau.

Supplementary Table 3. Participant characteristics by sex group: demographic, clinical, biomarker, and sleep architecture parameters (N=51)

	Female (N = 36)	Male (N = 15)	p value
**Sociodemographic and Clinical Characteristics**			
Age (years)	54 [9]**	64 [11]**	0.009
Education (years)	19 [6]	15 [7]	0.3
APOEε4+	10 (28%)	3 (20%)	0.8
MMSE	30 [1	29 [1]	0.061
Hypertension	4 (11%)*	7 (47%)*	0.015
Dyslipidemia	7 (19%)	6 (40%)	0.2
Diabetes	1 (2.8%)	1 (6.7%)	>0.9
Antidepressant Treatment	3 (8.3%)	1 (6.7%)	1
Hypnotic Treatment	4 (11.1%)	0 (0%)	0.24
BMI (kg/m2)	23.7 [5.4]	24.0 [3.0]	0.3
Time from NPSG to biofluid collection (days)	1 [9]	1 [3]	0.6
**Fluid Biomarkers**			
CSF YKL-40 (pg/mL)	161 [87]	232 [57]	0.008
CSF GFAP (pg/mL)	6,216 [3,563]	7,228 [4,976]	0.2
Plasma GFAP (pg/mL)	100 [88]	138 [89]	0.3
Plasma NfL (pg/mL)	6.8 [4.2]	9.1 [6.0]	0.092
CSF Aβ40	11,732 [4,601]	10,323 [1,697]	0.13
CSF Aβ42	1,199 [561]	1,035 [380]	0.094
CSF pTau181 (pg/mL)	34 [12]	32 [8]	0.7
CSF tTau (pg/mL)	256 [114]	243 [77]	0.5

	Female (N = 36)	Male (N = 15)	p value
**PSG Sleep Architecture Parameters**			
Total Sleep Time (minutes)	379 [61]*	333 [59*	0.038
Wake after sleep onset (minutes)	158 [93]	128 [75]	0.055
Sleep efficiency (%)	85 [10]*	73 [16]*	0.022
Sleep latency (min)	19 [23]	27 [15]	0.4
Stage N1 (min) / (%)	45 [36]/13 [12]	31 [50]/12 [16	0.9/0.6
Stage N2 (min) / (%)	187 [58]/51 [9]	157 [53]/48 [10]	0.057/0.5
Stage N3 (min) / (%)	73 [30]/20 [8]	72 [36]/19 [10]	0.3/0.5
Stage REM (min) / (%)	67 [25]/18 [5.2]	73 [34]/19.0 [9.0]	0.6 / 0.2
Number of stage shift	157 [63]	157 [69]	0.8
Number of Arousal	13 [10]	15 [12]	>0.9
Number of Awakening	19 [7]	24 [6]	0.084
Log10(Mean Slow Wave Activity)	222 [162]	228 [254]	0.8
**PSG Respiratory Parameters**			
OSA (AHI>5)	7 (19%)*	8 (53%)*	0.022
Moderate/Severe OSA (AHI>15)	3 (8.3%)	2 (13%)	0.6
AHI (events/hour)	3 [4]*	6 [8]*	0.046
T90 (%)	0.10 [0.43]	0.20 [1.40]	0.6
**Sleep questionnaire scores**			
PSQI	4 [3]	4 [4]	0.8
ESS	4 [6]	5 [4]	> 0.9
BQ (high risk)	2 (5.7%)	1 (6.7%)	> 0.9

Note: Values are presented as median [IQR] or n (%). Statistical comparisons were performed using the Wilcoxon rana-sum test for continuous variables and Fisher's exact test for categorical variables. Significance levels: p>0.05 (*), p<0.001 (**), p<0.0001 (***). Abbreviations: APOEε4+, carrier of one or two APOE ε4 alleles, MMSE, Mini-Mental state examination; BMI, body mass index; CSF, cerebrospinal fluid; YKL-40, chitinase-3-like protein 1; GFAP, glial fibrillary acidic protein; NfL, neurofilament light chain; Aβ40, Amyloid-β 1-40; Aβ42, Amyloid-β 1-42; pTau181, tau protein phosphorylated at threonine 181; tTau, total tau; NPSG, nocturnal polysomnography; AHI, apnea-hypopnea index; T90, time proportion with oxygen saturation below 90% during sleep; OSA, obstructive sleep apnea; PSQI, Pittsburgh Sleep Quality Index; ESS, Epworth Sleepiness Scale; BQ, Berlin Questionnaire.

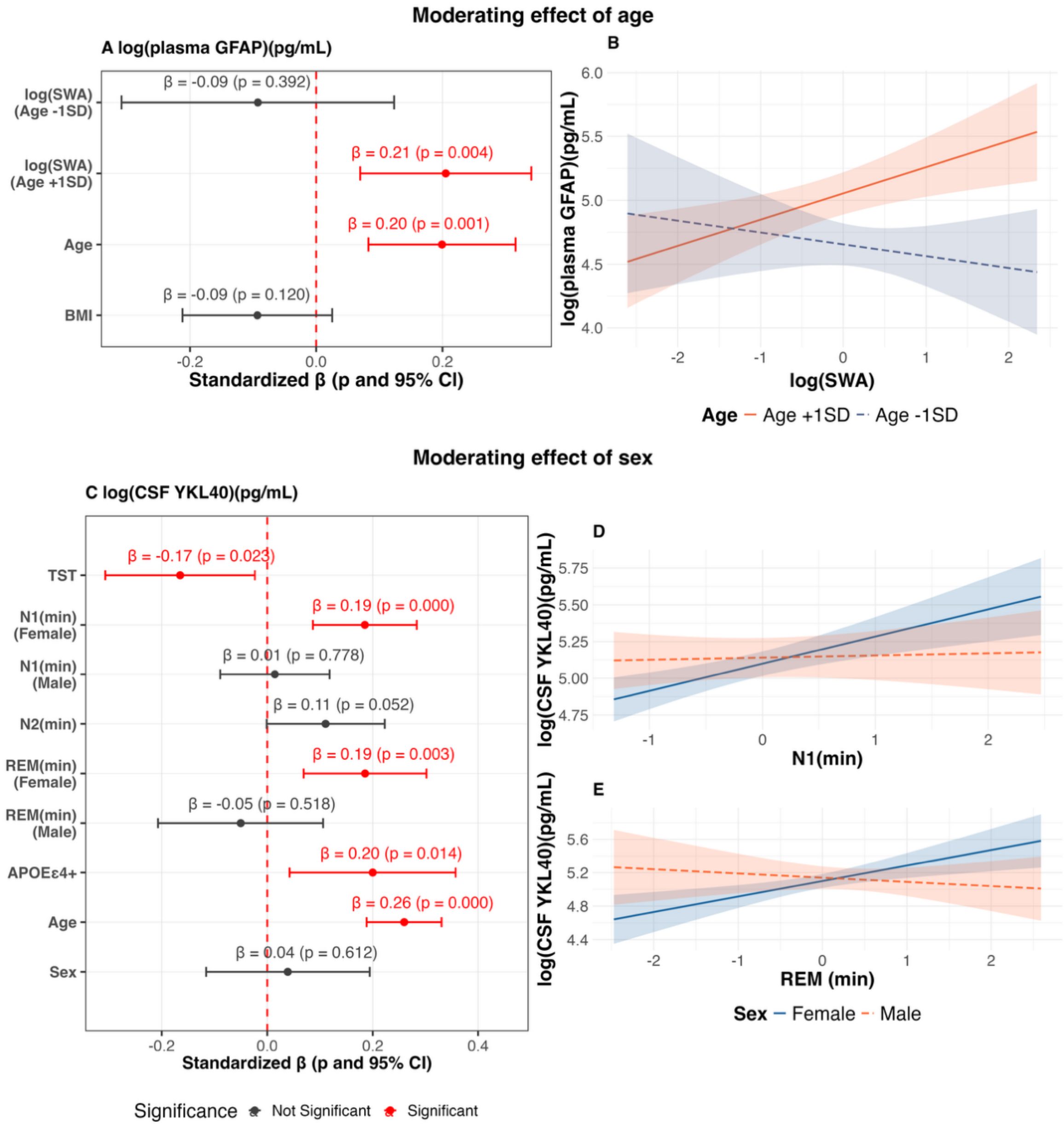

Supplementary Figure 1. Moderating effect of age and sex on the associations between sleep parameters and fluid biomarkers.

(Panels A and C, left) Forest plots showing standardized regression coefficients (dots indicating β), 95% confidence intervals (bars) and p-values from multivariate models including interaction terms between age or sex and sleep parameters, adjusted for age, sex, APOE genotype, BMI and OSA where appropriate. Statistically significant effects ($p<0.05$ and 95% CI not including zero) are shown in red. Biomarkers and skewed sleep parameters (e.g., SWA) were log-transformed prior to analysis (N = 47 for models with CSF biomarkers; N = 51 for models with plasma biomarkers).

(Panels B, D, and E, right) Interaction plots illustrating stratified associations between sleep parameters and biomarkers. For age, lines represent predicted associations at

mean age (green solid line), 1 SD below the mean (blue dashed line), and 1 SD above the mean (pink dashed line), with shaded areas indicating 95% confidence intervals. For sex, lines represent predicted associations for women (blue) and men (orange), with shaded areas indicating 95% confidence intervals.

Abbreviations: *log = logarithmic transformation; CSF = cerebrospinal fluid; GFAP = glial fibrillary acidic protein; YKL40 = chitinase-3-like protein 1; TST = total sleep time; N1(min) = N1 stage duration; N2(min) = N2 stage duration; N3(min) = N3 stage duration; REM(min) = REM stage duration; SWA = average slow-wave activity; APOE ε4+ = APOE ε4 carrier; BMI = body mass index.*

**Supplementary Table 4. Summary of moderation models by Age and Sex**				
	**RSE**	**Adjusted R2**	**F-statistic**	**p value**
**Age x SWA interaction on astrocytic fluid biomarkers**				
log(plasma GFAP)	0.3937	0.2836	5.75	0.0008
**Sex x sleep microarchitecture interaction on astrocytic fluid biomarkers**				
log(CSF YKL-40)	0.2131	0.6824	13.35	<0.0001
